# Development and validation of a machine learning model to predict postoperative complications following radical gastrectomy for gastric cancer

**DOI:** 10.3389/fonc.2025.1606938

**Published:** 2025-09-08

**Authors:** Zhenmeng Lin, Mingfang Yan, Hai Chen, Shenghong Wei, Yangming Li, Jinliang Jian

**Affiliations:** ^1^ Department of Gastrointestinal Surgery, Clinical Oncology School of Fujian Medical University and Fujian Cancer Hospital, Fuzhou, China; ^2^ Department of Anesthesiology, Clinical Oncology School of Fujian Medical University and Fujian Cancer Hospital, Fuzhou, China; ^3^ Department of Gastrointestinal Surgery, The First Hospital of Putian City, Putian, China

**Keywords:** gastric cancer, postoperative complications, machine learning, dynamic nomogram, surgery

## Abstract

**Objective:**

Postoperative complications significantly adversely affect recovery and prognosis following radical gastrectomy for gastric cancer. We developed and validated machine learning (ML) models to predict these complications and constructed a clinically applicable dynamic nomogram.

**Methods:**

Using a prospectively maintained database, we conducted a retrospective analysis of 1,486 patients from Fujian Cancer Hospital (training cohort) and 498 from the First Hospital of Putian City (validation cohort). Feature selection integrated Lasso regression, the Boruta algorithm, and Recursive Feature Elimination (RFE). Six ML models were developed and evaluated: TreeBagger (TB), Random Forest (RF), Support Vector Machine (SVM), Extreme Gradient Boosting (XGBoost), Gaussian Naïve Bayes (GNB), and Artificial Neural Network (ANN). The significant predictors identified were incorporated into a logistic regression model to determine independent risk factors, which then formed the basis of a dynamic nomogram deployed as an interactive web application for clinical use.

**Results:**

RF demonstrated numerically superior performance among the evaluated models in both cohorts. Independent risk factors included age, BMI, diabetes mellitus, ASA grade, operative time, and surgical approach. The dynamic nomogram achieved AUCs of 0.805 (training) and 0.856 (validation), with calibration curves and decision curve analysis confirming its reliability. DeLong’s test revealed no significant difference in AUC between the RF model and nomogram in either cohort (training: Z = -0.385, p = 0.701; validation: Z = -1.756, p = 0.058).

**Conclusion:**

While the RF model provided optimal predictive accuracy among ML algorithms, the interpretable nomogram offers comparable discrimination and clinical accessibility. Both tools facilitate the early identification of high-risk patients, enabling personalized interventions to optimize postoperative recovery.

## Introduction

Gastric cancer ranks as the fifth most common cancer globally and is the fourth leading cause of cancer-related death (1). Surgery remains the primary curative treatment; however, it poses significant challenges due to the complex anatomy of the stomach, its rich vascular supply, and the technical difficulty of lymph node dissection. Furthermore, reconstruction of the digestive tract alters normal anatomy, increasing both the likelihood and complexity of postoperative complications (2, 3).

Although perioperative mortality rates for gastric cancer have declined in recent decades, the incidence of postoperative complications remains substantial, ranging from 11.0% to 40.1% (4–8). These complications can significantly delay recovery, prolong hospitalization, increase healthcare costs, diminish quality of life, and adversely impact long-term survival (9, 10). Consequently, accurate preoperative risk assessment and early intervention represent a critical strategy for mitigating postoperative complications.

Machine learning (ML), a subset of artificial intelligence, leverages algorithms to uncover complex relationships within large datasets. Within healthcare, ML is increasingly employed to predict disease outcomes, personalize treatments, and enhance clinical decision-making, ultimately aiming to improve patient outcomes and optimize healthcare delivery (11, 12). However, existing predictive tools face significant limitations. Traditional scoring systems often oversimplify non-linear relationships, while many ML-based models exhibit methodological shortcomings, including reliance on small single-center cohorts, lack of external validation, and suboptimal handling of high-dimensional data during feature selection (13–17). Recent efforts to enhance clinical utility focus on ensemble methods (e.g., Random Forest, XGBoost) and interpretable nomograms. Nevertheless, comprehensive comparisons of multiple algorithms integrated with robust hybrid feature selection strategies remain lacking specifically for predicting complications following gastric cancer surgery. To address these critical gaps, we developed and validated six distinct ML models using a large multicenter cohort. Our approach integrates hybrid feature selection, rigorous external validation, and the development of a clinically accessible dynamic nomogram for practical implementation.

## Methods

### Patients

**Figure 1:**
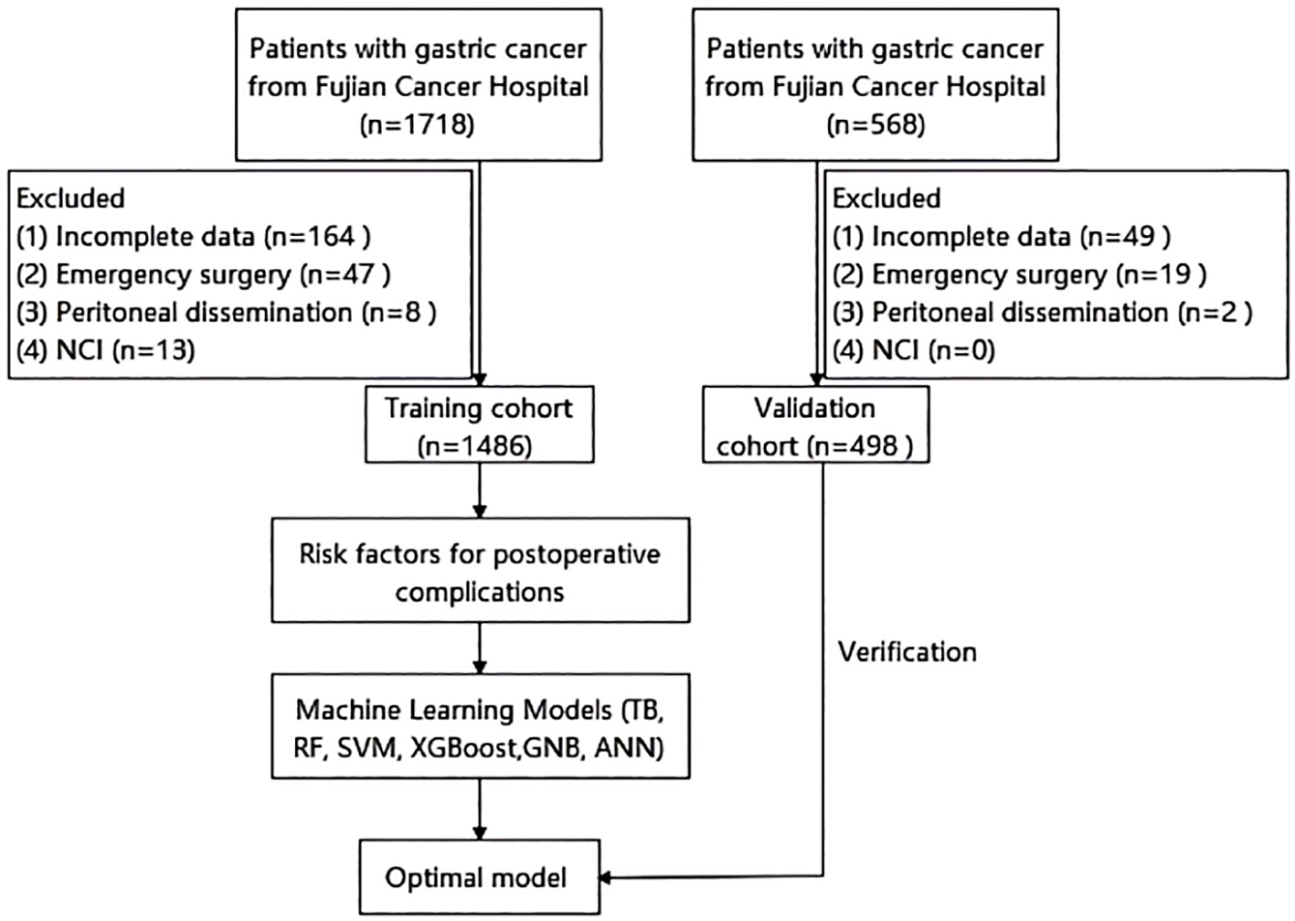
Flow chart of patient selection. NCI, neoadjuvant chemotherapy with immunotherapy; TB, TreeBagger; RF, random forest; SVM, support vector machine; XGBoost, extreme gradient boosting; GNB, Gaussian Naive Bayes; ANN, artificial neural network.

We performed a retrospective analysis using data from a prospectively maintained database. The analysis included 1,486 gastric cancer patients who underwent radical gastrectomy at Fujian Cancer Hospital between January 2020 and March 2024, constituting the training cohort. Inclusion criteria were: (1) age ≥ 18 years; (2) histologically confirmed gastric adenocarcinoma; and (3) radical gastrectomy. Exclusion criteria comprised: (1) incomplete clinical or pathological data; (2) emergency surgery; (3) intraoperative peritoneal dissemination; and (4) receipt of neoadjuvant chemotherapy with immunotherapy (NCI; excluded due to limited case numbers precluding meaningful subgroup analysis). An independent cohort of 498 patients from the First Hospital of Putian City, meeting identical inclusion criteria, served as the validation cohort. **Figure 1** illustrates the patient selection flowchart.

### Definitions and outcome measures

The severity of postoperative complications was assessed using the Clavien-Dindo classification system (18, 19), the standard grading system for surgical complications.

Age ≥ 65 was used as the threshold to define elderly patients, in line with previous clinical conventions (20, 21). Anemia was defined as hemoglobin <110 g/L in females and <120 g/L in males (22). Preoperative hypoalbuminemia was defined as serum albumin <35 g/L, a validated threshold associated with increased complication risk (23–25). and this threshold is commonly regarded as indicative of hypoalbuminemia. Body mass index (BMI) was categorized per WHO criteria: BMI < 18.5 kg/m² is underweight, BMI between 18.5 and 24.9 kg/m² is normal weight, and BMI ≥ 25 kg/m² is overweight (26–28). Tumor location is categorized into three regions—upper, middle, and lower third—based on the Japanese Gastric Cancer Treatment Guidelines (29).

### Implementation of machine learning models

All six models were implemented in Python 3.10 using scikit-learn (v1.3.0) and XGBoost (v1.7.5) with the following specifications:

TreeBagger (TB): 500 decision trees, bootstrap sampling, Gini impurity for splitting, and default scikit-learn parameters for other settings.

Random Forest (RF): 500 trees, Gini impurity criterion, max depth=10, min samples split=5.

Support Vector Machine (SVM): Radial basis function kernel (C = 1.0, gamma=‘scale’).

XGBoost: 300 estimators, learning rate=0.05, max depth=4, subsample=0.8.

Gaussian Naïve Bayes (GNB): Default scikit-learn parameters (priors adjusted to class distribution).

Artificial Neural Network (ANN): Single hidden layer (32 neurons), ReLU activation, Adam optimizer (learning rate=0.001).

All models underwent 5-fold stratified cross-validation on the training cohort.

### Statistical analysis

Categorical variables are presented as frequencies (percentages), with between-group comparisons using chi-square tests. Continuous variables are expressed as mean ± standard deviation (SD) or median [interquartile range, IQR] based on distribution normality, compared using independent t-tests or Mann-Whitney U tests as appropriate. Feature selection identified predictive variables through intersection analysis of three methods: Lasso regression, Boruta algorithm, and Recursive Feature Elimination (RFE). Model performance evaluation included: Receiver operating characteristic (ROC) curves with area under curve (AUC); Calibration curves; Decision curve analysis (DCA). Quantitative metrics: accuracy, sensitivity, specificity, positive predictive value (PPV), negative predictive value (NPV), and F1-score. Statistical significance was defined as P < 0.05.

## Results

### Patient characteristics and feature selection

In the training cohort, the overall incidence of postoperative complications was 20.5% (304/1,486), with 9.3% (138/1,486) classified as Clavien–Dindo grade ≥ IIIa; in the validation cohort, these rates were 23.9% (119/498) and 11.6% (58/498), respectively (**Supplementary Table 1, 2**). The specific types and frequencies of postoperative complications are detailed in **Table 1**. Baseline characteristics were comparable between cohorts except for sex, drinking history, dyslipidemia, and CA19-9 levels (**Table 2**).

**Table 1:** Types and incidence of postoperative complications in the training and validation cohorts.

Complication Type	Training cohort (n=1486)	Validation cohort (n=498)	p
Overall complication	304 (20.5)	119 (23.9)	0.105
Pulmonary infection	176 (11.8)	59 (11.8)	0.998
Surgical incision infection	11 (0.7)	7 (1.4)	0.175
Anastomotic leakage	68 (4.6)	33 (6.6)	0.072
Anastomotic stricture	29 (2.0)	11 (2.2)	0.724
Intestinal obstruction	75 (5.0)	29 (5.8)	0.501
Abdominal infection	86 (5.8)	34 (6.8)	0.399
Hemorrhage	27 (1.8)	18 (3.6)	0.020
Lymphorrhea	48 (3.2)	11 (2.2)	0.246
Urinary tract infection	6 (0.4)	0 (0)	0.156
Other	38 (2.6)	11 (2.2)	0.665

**Table 2:** Comparison of clinicopathological characteristics between training and validation cohorts.

Variable	Training cohort (n=1486)	Validation cohort (n=498)	p
Age (years)			0.471
<65	854 (57.5)	277 (55.6)	
≥65	632 (42.5)	221 (44.4)	
Sex			0.018
Male	986 (66.4)	359 (72.1)	
Female	500 (33.6)	139 (27.9)	
BMI (kg/m^2^)			0.519
underweight	92 (6.2)	38 (7.6)	
normal	1017 (68.4)	338 (67.9)	
overweight	377 (25.4)	122 (24.5)	
Previous abdominal surgery			0.174
Yes	261 (17.6)	101 (20.3)	
No	1225 (82.4)	397 (79.7)	
Drinking history			0.022
Yes	412 (27.7)	112 (22.5)	
No	1074 (72.3)	386 (77.5)	
Smoking history			0.610
Yes	365 (24.6)	128 (25.7)	
No	1121 (75.4)	370 (74.3)	
Diabetes mellitus			0.284
Yes	1191 (80.1)	388 (77.9)	
No	295 (19.9)	110 (22.1)	
Hypertension			0.366
Yes	387 (26.0)	140 (28.1)	
No	1099 (74.0)	358 (71.9)	
Dyslipidemia			0.029
Yes	354 (23.8)	143 (28.7)	
No	1132 (76.2)	355 (71.3)	
Tumor location			0.296
Upper-third	392 (26.4)	146 (29.3)	
Middle-third	464 (31.2)	159 (31.9)	
Lower-third	630 (42.4)	193 (38.8)	
FEV1/FVC, %, median (IQR)	72.00 [69.00, 73.00]	72.00 [69.00, 74.00]	0.108
Tumor size (cm)			0.545
<5	808 (54.4)	263 (52.8)	
≥5	678 (45.6)	235 (47.2)	
ASA grade			0.235
I	1050 (70.7)	334 (67.1)	
II	291 (19.6)	104 (20.9)	
III	145 (9.8)	60 (12.0)	
Neoadjuvant chemotherapy			0.126
Yes	395 (26.6)	150 (30.1)	
No	1091 (73.4)	348 (69.9)	
Multivisceral resection			0.399
Yes	86 (5.8)	34 (6.8)	
No	1400 (94.2)	464 (93.2)	
Preoperative anemia			0.255
Yes	461 (31.0)	141 (28.3)	
No	1025 (69.0)	357 (71.7)	
Preoperative hypoalbuminemia			0.236
Yes	346 (23.3)	129 (25.9)	
No	1140 (76.7)	369 (74.1)	
Preoperative WBC,×10^9^/L, median (IQR)	6.50 [5.40, 7.50]	6.30 [5.30, 7.30]	0.193
Preoperative BUN, mg/dl, median (IQR)	6.45 [5.67, 7.34]	6.67 [5.75, 7.34]	0.323
Preoperative total bilirubin, mg/dl, median (IQR)	12.90 [9.90, 16.90]	13.50 [10.80, 17.30]	0.089
CEA, ng/ml			0.243
<5	1191 (80.1)	387 (77.7)	
≥5	295 (19.9)	111 (22.3)	
CA19-9, U/ml			0.036
<30	1270 (85.5)	406 (81.5)	
≥30	216 (14.5)	92 (18.5)	
Surgical approach			0.828
Open	437 (29.4)	149 (29.9)	
Laparoscopic	1049 (70.6)	349 (70.1)	
Operation time (h)			0.604
<3	754 (50.7)	246 (49.4)	
≥3	732 (49.3)	252 (50.6)	
Estimated blood loss (ml)			0.505
<200	679 (45.7)	219 (44.0)	
≥200	807 (54.3)	279 (56.0)	
Histological type			0.333
Well/Moderately	363 (24.4)	111 (22.3)	
Poorly/Undifferentiated	1123 (75.6)	387 (77.7)	
Type of operation			0.175
Distal gastrectomy	849 (57.1)	307 (61.6)	
Proximal gastrectomy	76 (5.1)	26 (5.2)	
Total gastrectomy	561 (37.8)	165 (33.1)	
Extent of lymph node dissection			0.092
<D2	175 (11.8)	45 (9.0)	
≥ D2	1311 (88.2)	453 (91.0)	
Intraoperative blood transfusion			0.250
Yes	260 (17.5)	76 (15.3)	
No	1226 (82.5)	422 (84.7)	
Reconstruction method			0.610
Intracorporeal	155 (10.4)	56 (11.2)	
Extracorporeal	1331 (89.6)	442 (88.8)	
Number of removed lymph nodes, mean (SD)	33.1 ± 13.0	32.3 ± 12.6	0.269
Pathological stage			0.587
I	349 (23.5)	113 (22.7)	
II	251 (16.9)	76 (15.3)	
III	886 (59.6)	309 (62.0)	

BMI, body mass index; ASA, American society of anesthesiologists classification; WBC, white blood cell; BUN, blood urea nitrogen; CEA, carcinoembryonic antigen; CA19-9, cancer antigen 19-9; IQR, interquartile range; SD, standard deviation.

**Figure 2:**
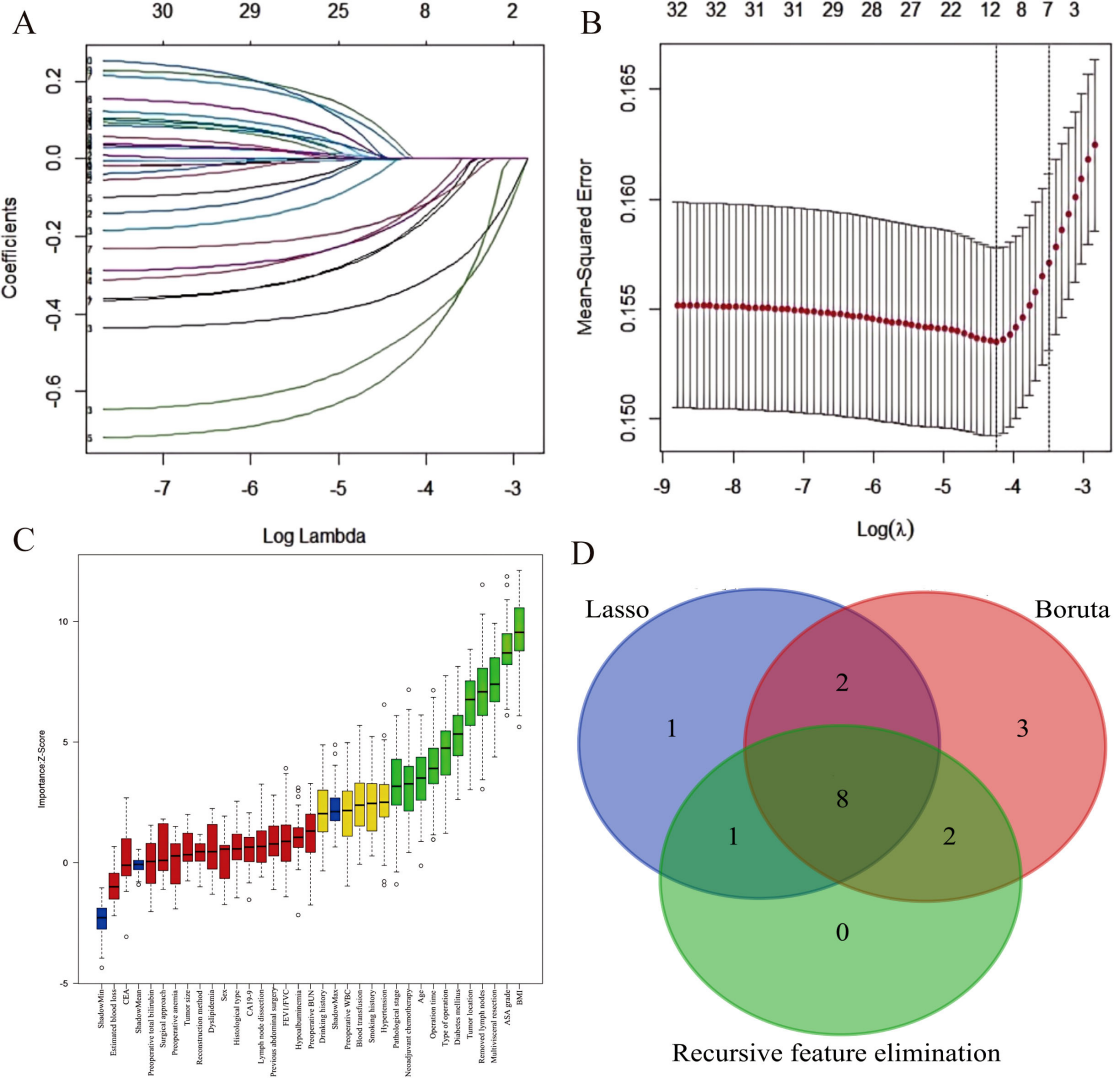
Feature selection for postoperative complication risk factors. **(A)** LASSO coefficient path showing shrinkage of 12 predictors. **(B)** Five-fold cross-validated deviance curve identifying the optimal model. **(C)** Boruta-derived importance plot highlighting 15 key predictors. **(D)** Venn diagram demonstrating convergence of three feature-selection methods on eight final predictors.

Through intersection analysis of three feature selection methods (Lasso regression, Boruta algorithm, and RFE), we identified eight factors associated with postoperative complications in the training cohort: age, BMI, diabetes mellitus, ASA grade, neoadjuvant chemotherapy, multivisceral resection, operative time, and surgical approach (**Figure 2**).

### Establishment and comparison of machine learning models

**Figure 3:**
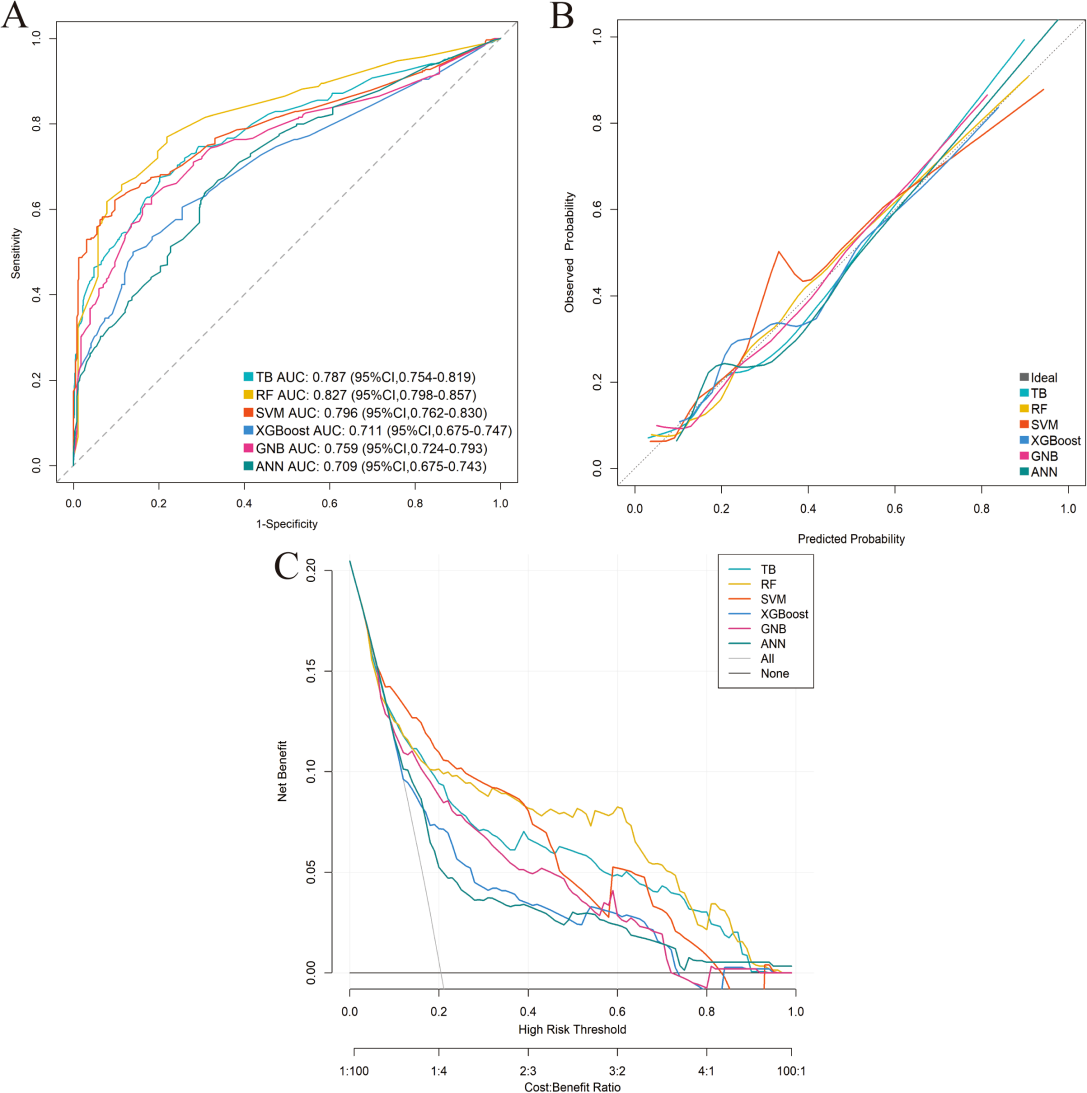
Performance comparison of the six machine-learning models in the training cohort. **(A)** ROC curve. **(B)** Calibration curve. **(C)** DCA.

**Figure 4:**
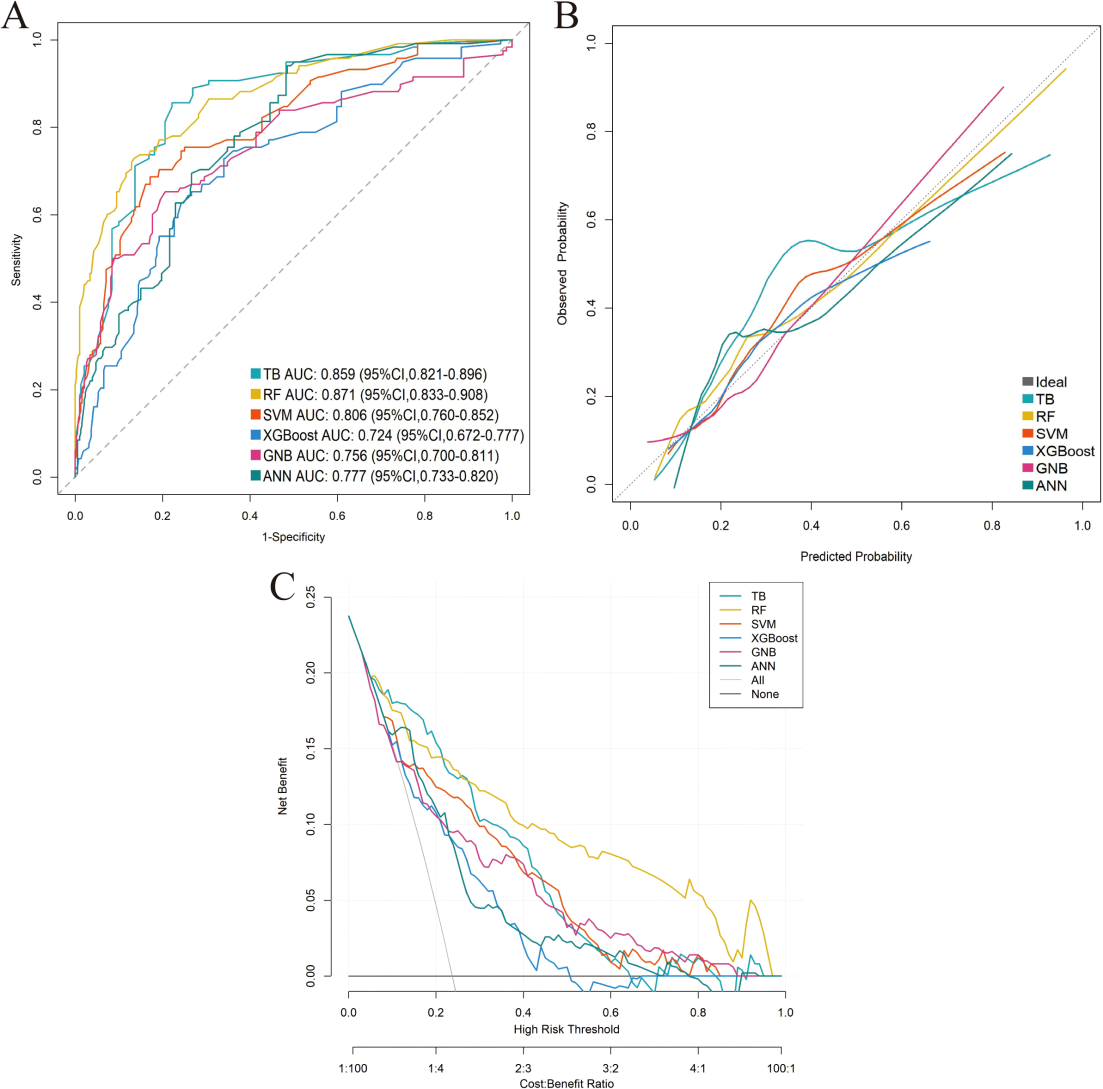
Performance comparison of the six machine-learning models in the external validation cohort. **(A)** ROC curve. **(B)** Calibration curve. **(C)** DCA.

Six distinct ML models (TB, RF, SVM, XGBoost, GNB, ANN) were developed using the training cohort to predict postoperative complications. Model performance was rigorously assessed via five-fold cross-validation. Among the evaluated models, RF demonstrated superior predictive performance in the training cohort across key metrics including AUC, sensitivity, and NPV (**Table 3**; **Supplementary Figure 1**; **Figures 3A–C**). This performance advantage was maintained in the independent validation cohort (**Supplementary Table 3**; **Supplementary Figure 2**; **Figures 4A–C**), establishing RF as the optimal model among the tested algorithms.

**Table 3:** Performance of ML models in training cohort.

Model	AUC (95%CI)	Accuracy (95%CI)	Sensitivity (95%CI)	Specificity (95%CI)	PPV (95%CI)	NPV (95%CI)	F1 score (95%CI)
TB	0.787 (0.754-0.819)	0.773 (0.731-0.807)	0.674 (0.632-0.711)	0.798 (0.767-0.829)	0.462 (0.433-0.489)	0.905 (0.872-0.938)	0.548 (0.540-0.581)
RF	0.827 (0.798-0.857)	0.779 (0.739-0.809)	0.770 (0.738-0.803)	0.781 (0.748-0.814)	0.475 (0.429-0.519)	0.930 (0.894-0.970)	0.588 (0.531-0.628)
SVM	0.796 (0.762-0.830)	0.845 (0.803-0.882)	0.622 (0.583-0.672)	0.903 (0.868-0.949)	0.622 (0.573-0.668)	0.903 (0.853-0.942)	0.622 (0.587-0.653)
XGBoost	0.711 (0.675-0.747)	0.787 (0.761-0.813)	0.500 (0.437-0.539)	0.860 (0.832-0.895)	0.479 (0.432,0.520)	0.870 (0.834-0.902)	0.490 (0.449-0.528)
GNB	0.759 (0.724-0.793)	0.779 (0.748-0.816)	0.628 (0.592-0.661)	0.817 (0.787-0.841)	0.469 (0.428-0.503)	0.895 (0.863-0.923)	0.536 (0.507-0.567)
ANN	0.709 (0.675-0.743)	0.680 (0.638-0.710)	0.641 (0.606-0.679)	0.690 (0.653-0.721)	0.348 (0.309-0.397)	0.882 (0.838-0.908)	0.452 (0.421-0.478)

### Nomogram construction and validation

**Figure 5:**
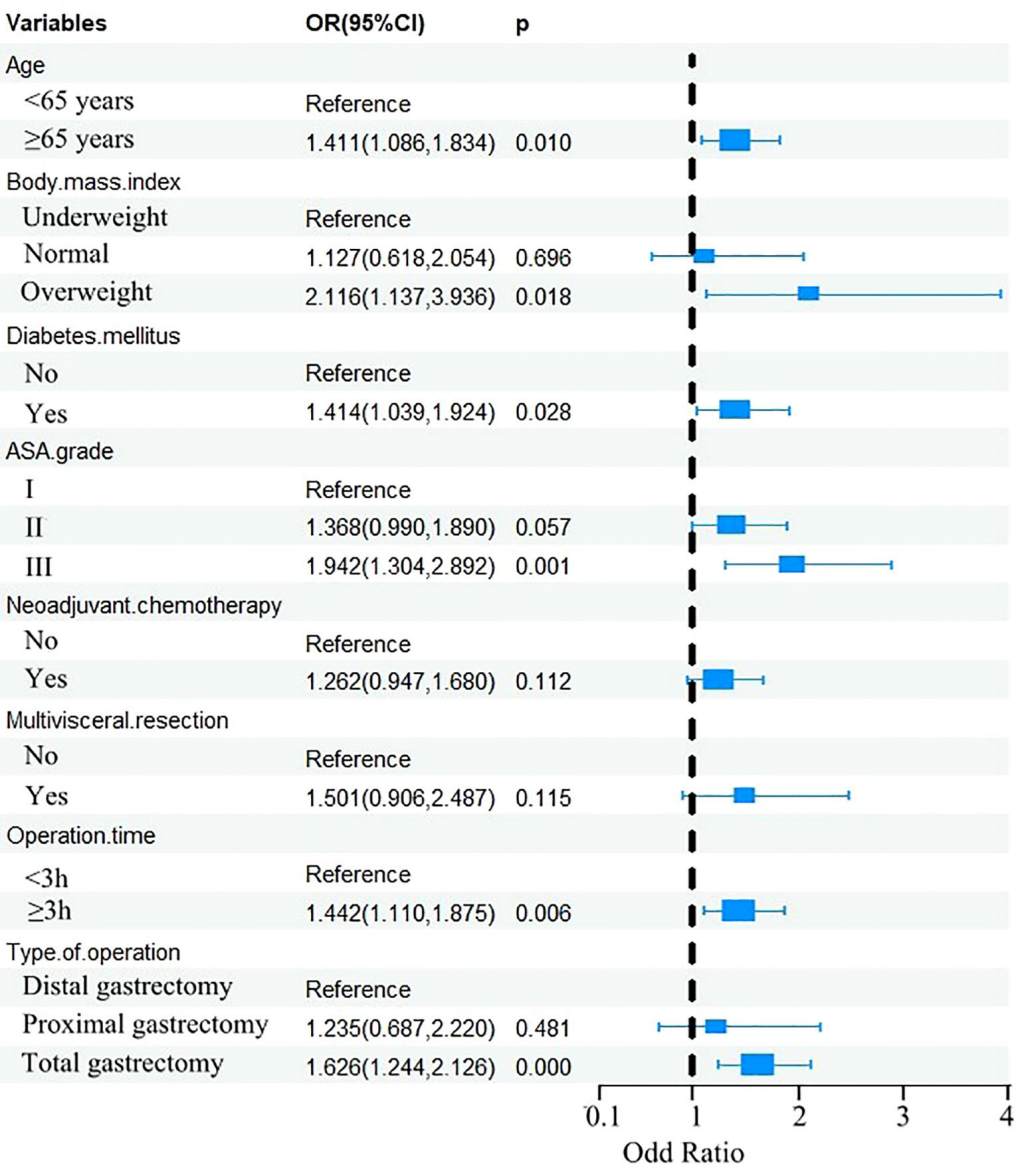
Forest plot of multivariable logistic regression for postoperative complications.

**Figure 6:**
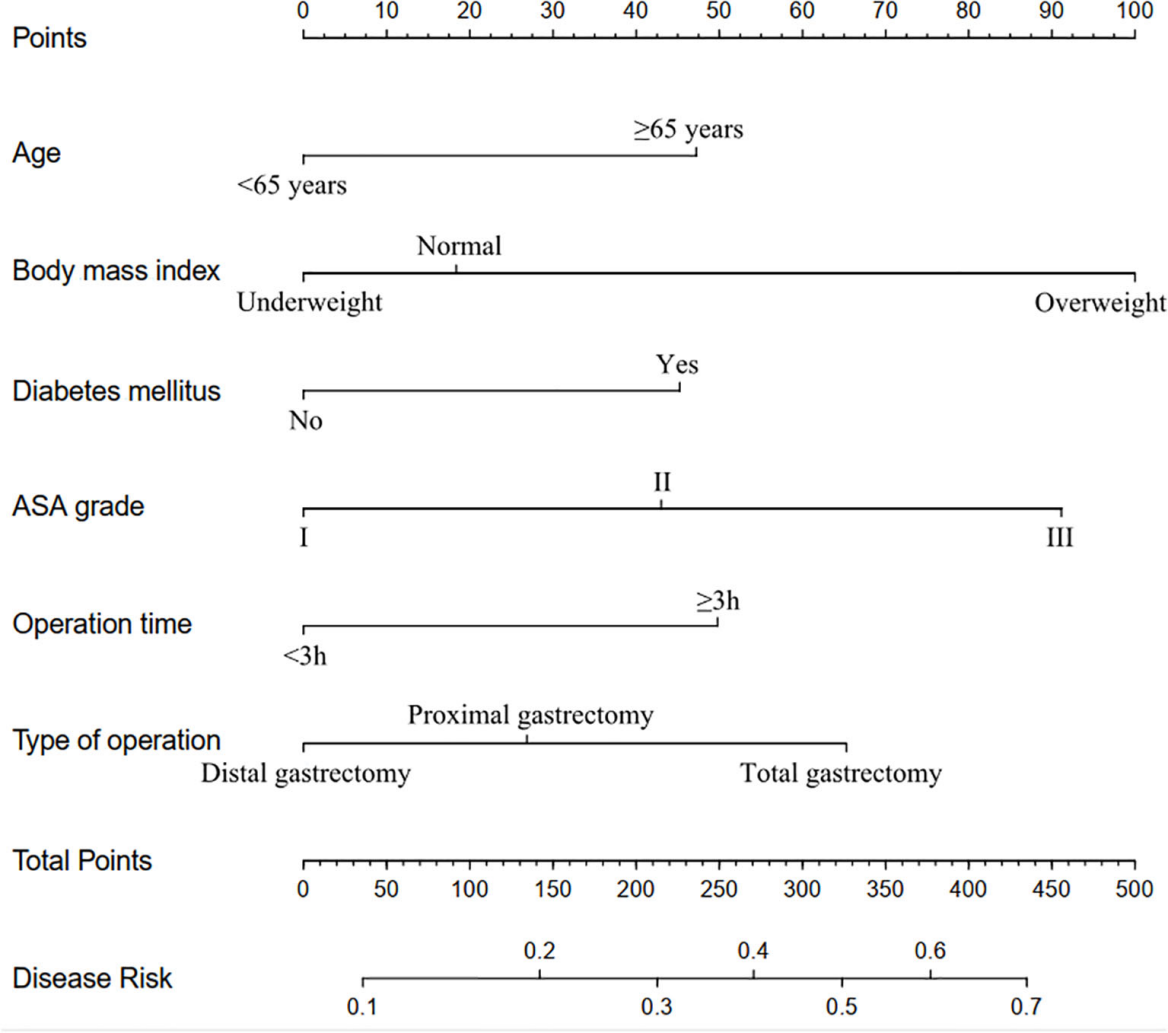
Nomogram for predicting the risk of postoperative complications.

The eight factors identified via intersection analysis of three feature selection methods (Lasso regression, Boruta algorithm, and RFE) were incorporated into a multivariable logistic regression model to screen for independent risk factors for postoperative complications, which revealed that age, BMI, diabetes mellitus, ASA grade, operative time, and surgical approach were independently associated with postoperative complications (**Figure 5**). A nomogram and its dynamic version were developed to facilitate clinical application, with the dynamic tool accessible via a web application (https://lzmdoc123456789.shinyapps.io/pcingc) (**Figures 6**). Internal validation of the nomogram demonstrated excellent calibration, as evidenced by the calibration curve showing close alignment between predicted and observed outcomes.

**Figure 7:**
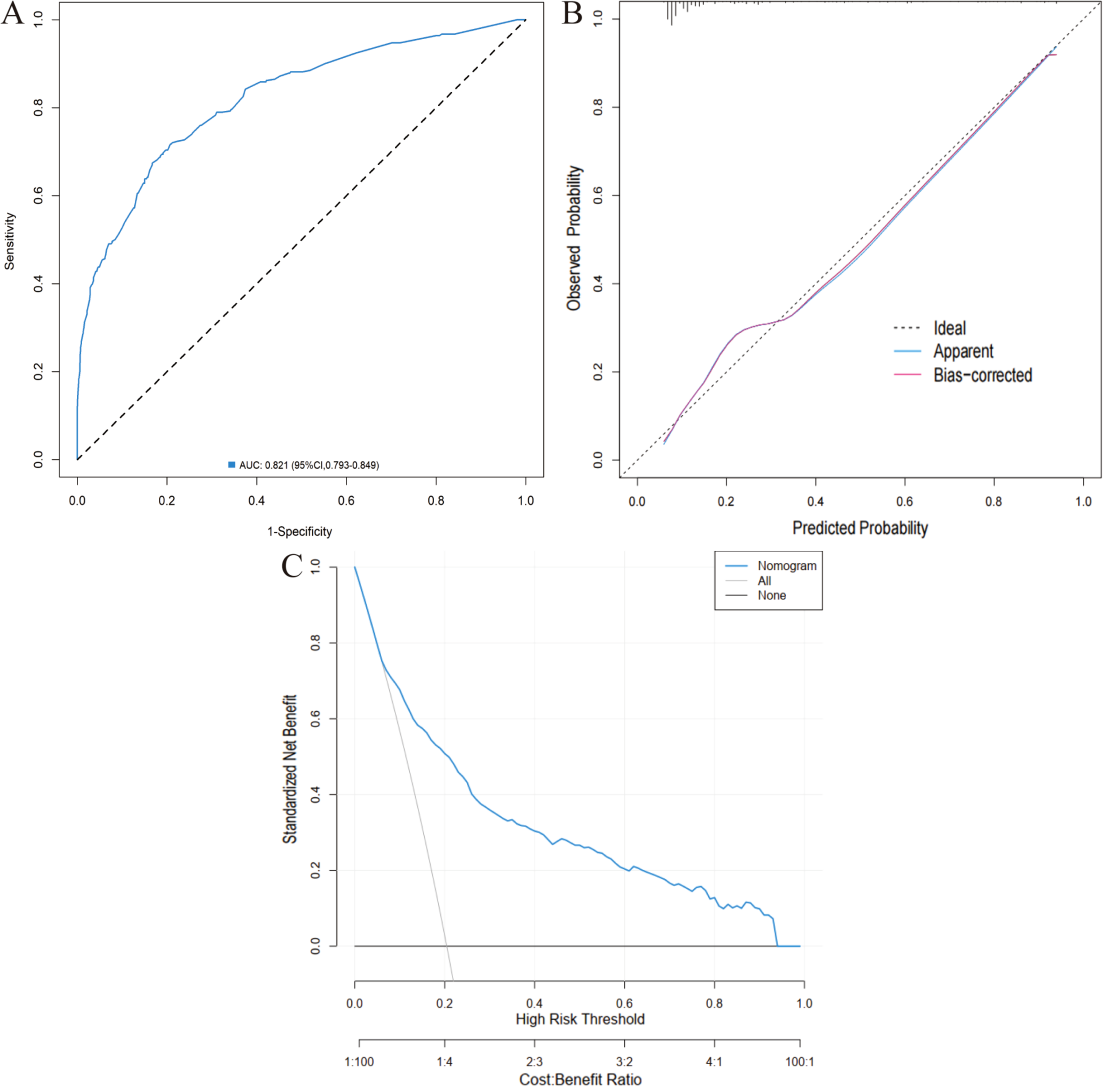
Validation of the nomogram in the training cohort. **(A)** ROC curve. **(B)** Calibration curve. **(C)** DCA.

Additionally, DCA confirmed its clinical utility, with favorable net benefits across threshold probabilities ranging from 0.06 to 0.95 (**Figure 7**).

**Figure 8:**
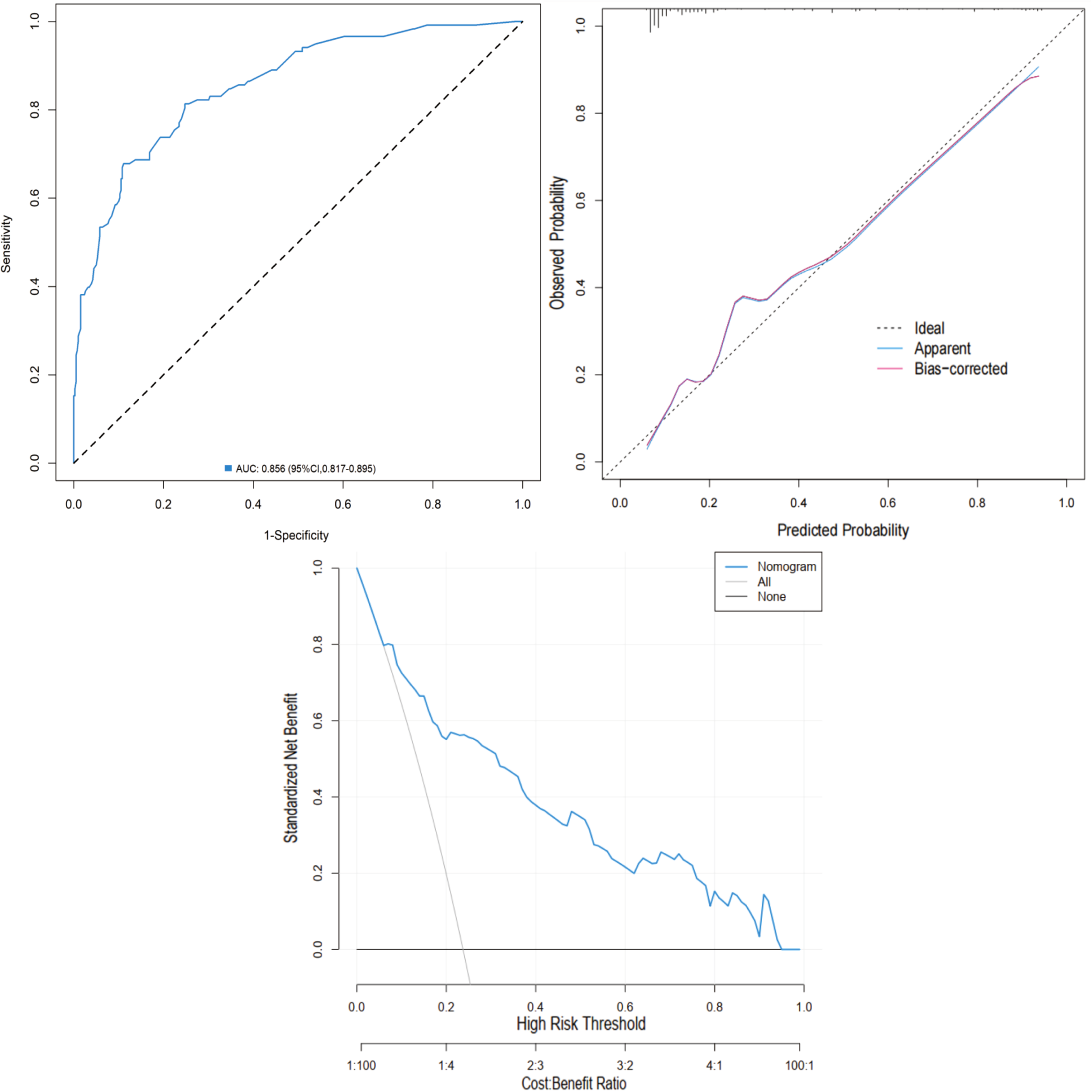
Validation of the nomogram in the validation cohort. **(A)** ROC curve. **(B)** Calibration curve. **(C)** DCA.

External validation was performed using the validation cohort. ROC curve analysis yielded an AUC of 0.856 (95% CI: 0.817-0.895), indicating excellent discriminatory ability. The calibration curve further confirmed the model’s accuracy, with predicted outcomes closely aligning with observed results. Moreover, DCA demonstrated favorable net benefits across a broad range of threshold probabilities (0.04 to 0.97), supporting the nomogram’s clinical value (**Figure 8**).

### Comparison of predictive performance between RF model and nomogram

To compare the optimal ML model (RF) with the nomogram, DeLong’s test was used to analyze their ROC curves. The RF model showed marginally higher AUCs than the nomogram in both cohorts (training: 0.827 vs. 0.805; validation: 0.871 vs. 0.856), but these differences were not statistically significant (training: Z = -0.385, p = 0.701; validation: Z = -1.756, p = 0.058).

## Discussion

Postoperative complications following gastric cancer surgery pose substantial threats to patient recovery and long-term survival. These adverse events significantly elevate mortality risk, prolong hospitalization duration, escalate healthcare expenditures, and impair quality of life - particularly concerning for cancer patients requiring adjuvant therapy. Such complications may also compromise functional recovery and nutritional status, creating barriers to timely oncological treatment (30, 31). In this study, the incidence of postoperative complications was 20.5% in the training cohort and 23.9% in the validation cohort, which aligns with findings from previous studies on gastric cancer surgery. Kanda et al. (32) reported a postoperative complication rate of 22.5%. Similarly, a study analyzing 663 gastric cancer patients found that 20.8% experienced postoperative complications (7). Data from the National Clinical Database, which includes over 33,917 Japanese patients, also supports this finding, showing a complication rate of 18.3% (33). However, other studies report higher complication rates. A European observational, retrospective trial indicated that 33% of patients experienced at least one postoperative complication (34), and a randomized controlled trial (JCOG1001) found a complication rate of 34.3% (4). The variation in postoperative complication rates may be attributed to differences in the definition of complications, patient population characteristics (such as age and comorbidities), surgical techniques, the experience of the surgical team, and postoperative care protocols.

ML has proven valuable for improving predictive accuracy in clinical settings (35–37). In the present study, among the six evaluated machine learning algorithms, the RF model showed numerically superior performance, achieving the highest AUC in both cohorts. However, DeLong’s test revealed no statistically significant difference in discriminative ability between the optimal RF model and the logistic regression-based nomogram. This comparable predictive performance, combined with the nomogram’s visual interpretability and user-friendly design, underscores its value as a pragmatic clinical tool. The dynamic nomogram facilitates rapid point-of-care risk stratification without requiring computational expertise, enabling clinicians to intuitively assess risk factors and implement personalized preventive strategies for high-risk patients. Our findings support a dual-model approach: the RF model serves as a high-performance reference standard in settings with adequate computational resources, while the dynamic nomogram offers an immediately deployable alternative with preserved discriminative power. This strategy balances algorithmic performance with real-world applicability across diverse healthcare contexts.

In this study, elderly patients were found to be more prone to postoperative complications. As individuals age, various physiological changes occur, including a decline in organ function, slower wound healing, and a weakened immune response, all of which complicate recovery. Moreover, elderly patients often have multiple comorbidities, which further delay the recovery process. Additionally, aging is frequently associated with sarcopenia, frailty, and a reduction in muscle mass and strength, all of which diminish the body’s ability to tolerate surgical and anesthetic stressors (38–40).

Consistent with previous studies (41–43), our study confirmed overweight as an independent risk factor for postoperative complications. Excess body weight contributes to increased intra-abdominal pressure, leading to impaired tissue oxygenation and impaired wound healing (44). Furthermore, individuals with higher body weight are more likely to develop comorbid conditions (e.g., diabetes mellitus), thereby exacerbating the risk of complications. Additionally, adiposity is associated with a chronic inflammatory state, which may compromise immune function and predispose patients to infections and prolonged inflammation (45). Lastly, overweight patients often undergo longer operative times and more complex surgical procedures—both well-established factors that increase the likelihood of postoperative complications (46).

Our study corroborates previous findings that diabetes mellitus significantly increases the risk of postoperative adverse events (13, 47). Hyperglycemia in diabetic patients impairs immune function by disrupting neutrophil chemotaxis and phagocytosis (48). Elevated blood glucose levels also impair collagen synthesis and extracellular matrix remodeling, both crucial to tissue repair, thereby contributing to poor wound healing (49). Moreover, diabetes is often associated with microvascular changes (e.g., impaired circulation), which can further compromise tissue oxygenation and delay healing (50).

The ASA physical status classification system is a widely utilized tool for evaluating preoperative patient health status (51). In our study, patients classified as ASA III had a higher likelihood of developing adverse postoperative outcomes relative to those with lower ASA grades. Severe comorbidities commonly observed in ASA III patients—such as cardiovascular disease, diabetes, and respiratory disorders—reduce their physiological reserves, thereby impairing tolerance to surgical stress. Furthermore, such patients with severe systemic diseases are at increased risk of infection due to impaired immune function, as these conditions often induce a chronic inflammatory state and impair immune responses (18, 52).

Our study confirmed operative time as an independent risk factor for postoperative complications. Prolonged operative time is frequently linked to more complex surgical procedures, increased tissue trauma, and extended anesthesia exposure—all of which increase the incidence of complications, including infections, bleeding, and delayed recovery (53, 54).

We identified total gastrectomy as a significant risk factor for postoperative complications. Total gastrectomy entails more extensive lymph node dissection and removal of a larger volume of tissue, rendering it a more invasive procedure that often results in greater surgical trauma and a higher risk of postoperative complications. Esophagojejunal anastomosis, a key step in gastrointestinal reconstruction after total gastrectomy, involves deep anastomosis in a confined surgical space, making it both complex and challenging. Specifically, in patients with excessive visceral fat, tension or stretching of the anastomosis may induce serosal tears and bleeding (19, 55).

This study has several limitations. First, despite using a prospectively maintained database, it is a retrospective analysis, and certain parameters (e.g., preoperative nutritional status) that may influence postoperative complications (56–58), were not available. Second, the role of NCI in gastric cancer is under active investigation and has shown promising results (59–61); however, due to the limited number of cases at our center, NCI was excluded from this study. Third, external validation was performed at only one hospital, which could restrict the generalizability of our findings.

## Conclusions

This study developed and validated ML models to predict postoperative complications in gastric cancer patients undergoing radical gastrectomy. Among the six evaluated models, RF model demonstrated numerically superior performance, while a logistic regression-based nomogram-incorporating key predictors including age, BMI, diabetes mellitus, ASA grade, operative time, and surgical approach-exhibited comparable discriminative ability and clinical practicability. Both tools facilitate the identification of high-risk patients and can guide clinical decision-making to optimize postoperative outcomes.

## Data Availability

The original contributions presented in the study are included in the article/**Supplementary Material**. Further inquiries can be directed to the corresponding author.

## References

[1] Sung H Ferlay J Siegel RL Laversanne M Soerjomataram I Jemal A. Global cancer statistics 2020: GLOBOCAN estimates of incidence and mortality worldwide for 36 cancers in 185 countries. CA Cancer J Clin. (2021) 71:209–49. doi: 10.3322/caac.21660, PMID: 33538338

[2] Terashima M. The 140 years’ journey of gastric cancer surgery: From the two hands of Billroth to the multiple hands of the robot. Ann Gastroenterol Surg. (2021) 5:270–7. doi: 10.1002/ags3.12442, PMID: 34095716 PMC8164465

[3] Polkowski WP Gęca K Skórzewska M. How to measure quality of surgery as a component of multimodality treatment of gastric cancer. Ann Gastroenterol Surg. (2024) 8:740–9. doi: 10.1002/ags3.12833, PMID: 39229566 PMC11368491

[4] Tokunaga M Kurokawa Y Machida R Sato Y Takiguchi S Doki Y. Impact of postoperative complications on survival outcomes in patients with gastric cancer: exploratory analysis of a randomized controlled JCOG1001 trial. Gastric Cancer. (2021) 24:214–23. doi: 10.1007/s10120-020-01102-3, PMID: 32601909

[5] Kurokawa Y Doki Y Mizusawa J Terashima M Katai H Yoshikawa T. Bursectomy versus omentectomy alone for resectable gastric cancer (JCOG1001): a phase 3, open-label, randomised controlled trial. Lancet Gastroenterol Hepatol. (2018) 3:460–8. doi: 10.1016/S2468-1253(18)30090-6, PMID: 29709558

[6] Lee JH Park DJ Kim HH Lee HJ Yang HK. Comparison of complications after laparoscopy-assisted distal gastrectomy and open distal gastrectomy for gastric cancer using the Clavien-Dindo classification. Surg Endosc. (2012) 26:1287–95. doi: 10.1007/s00464-011-2027-0, PMID: 22044981

[7] Yu F Huang C Cheng G Xia X Zhao G Cao H. Prognostic significance of postoperative complication after curative resection for patients with gastric cancer. J Cancer Res Ther. (2020) 16:1611–6. doi: 10.4103/jcrt.JCRT_856_19, PMID: 33565507

[8] Nakazawa N Sohda M Yamaguchi A Watanabe T Saito H Ubukata Y. Preoperative risk factors and prognostic impact of postoperative complications associated with total gastrectomy. Digestion. (2022) 103:397–403. doi: 10.1159/000525356, PMID: 35724642

[9] Yu H Xu L Yin S Jiang J Hong C He Y. Risk factors and prognostic impact of postoperative complications in patients with advanced gastric cancer receiving neoadjuvant chemotherapy. Curr Oncol. (2022) 29:6496–507. doi: 10.3390/curroncol29090511, PMID: 36135080 PMC9498105

[10] Han WH Oh YJ Eom BW Yoon HM Kim YW Ryu KW. Prognostic impact of infectious complications after curative gastric cancer surgery. Eur J Surg Oncol. (2020) 46:1233–8. doi: 10.1016/j.ejso.2020.04.032, PMID: 32362466

[11] Painuli D Bhardwaj S Köse U. Recent advancement in cancer diagnosis using machine learning and deep learning techniques: A comprehensive review. Comput Biol Med. (2022) 146:105580. doi: 10.1016/j.compbiomed.2022.105580, PMID: 35551012

[12] Singh AK Ling J Malviya R. Prediction of cancer treatment using advancements in machine learning. Recent Pat Anticancer Drug Discov. (2023) 18:364–78. doi: 10.2174/1574892818666221018091415, PMID: 36263487

[13] Liu ZK Ma WX Zhang JJ Liu SD Duan XL Wang ZZ. Risk factor analysis and establishment of a predictive model for complications of elderly advanced gastric cancer with Clavien-Dindo classification ≥ II grade. BMC Cancer. (2024) 24:1185. doi: 10.1186/s12885-024-12965-5, PMID: 39333976 PMC11437802

[14] Lan Q Guan X Lu S Yuan W Jiang Z Lin H. Radiomics in addition to computed tomography-based body composition nomogram may improve the prediction of postoperative complications in gastric cancer patients. Ann Nutr Metab. (2022) 78:316–27. doi: 10.1159/000526787, PMID: 36041416

[15] Chen X Zhang W Sun X Shi M Xu L Cai Y. Metabolic syndrome predicts postoperative complications after gastrectomy in gastric cancer patients: Development of an individualized usable nomogram and rating model. Cancer Med. (2020) 9:7116–24. doi: 10.1002/cam4.3352, PMID: 33470549 PMC7541147

[16] Lu S Yan M Li C Yan C Zhu Z Lu W. Machine-learning-assisted prediction of surgical outcomes in patients undergoing gastrectomy. Chin J Cancer Res. (2019) 31:797–805. doi: 10.21147/j.issn.1000-9604.2019.05.09, PMID: 31814683 PMC6856706

[17] Hong QQ Yan S Zhao YL Fan L Yang L Zhang WB. Machine learning identifies the risk of complications after laparoscopic radical gastrectomy for gastric cancer. World J Gastroenterol. (2024) 30:79–90. doi: 10.3748/wjg.v30.i1.79, PMID: 38293327 PMC10823896

[18] Lian B Chen J Li Z Ji G Wang S Zhao Q. Risk factors and clavien-dindo classification of postoperative complications after laparoscopic and open gastrectomies for gastric cancer: A single-center, large sample, retrospective cohort study. Cancer Manag Res. (2020) 12:12029–39. doi: 10.2147/CMAR.S275621, PMID: 33262653 PMC7700075

[19] Lin Z Yan M Lin Z Xu Y Zheng H Peng Y. Short-term outcomes of distal gastrectomy versus total gastrectomy for gastric cancer under enhanced recovery after surgery: a propensity score-matched analysis. Sci Rep. (2024) 14:17594. doi: 10.1038/s41598-024-68787-9, PMID: 39080478 PMC11289314

[20] Yan M Lin Z Zheng H Lai J Liu Y Lin Z. Development of an individualized model for predicting postoperative delirium in elderly patients with hepatocellular carcinoma. Sci Rep. (2024) 14:11716. doi: 10.1038/s41598-024-62593-z, PMID: 38777824 PMC11111779

[21] Niu P Zhang F Ma D Zhou X Zhu Y Luan X. Trends of older gastric cancer incidence, mortality, and survival in the highest gastric cancer risk area in China: 2010-2019 and prediction to 2024. BMC Public Health. (2024) 24:2449. doi: 10.1186/s12889-024-19944-2, PMID: 39251980 PMC11382508

[22] Society of Chemotherapy CAA Committee of Neoplastic Supportive-Care CAA. Consensus on the clinical diagnosis, treatment, and prevention of cancer related anemia in China (2023 edition). Zhonghua Zhong Liu Za Zhi. (2023) 45:1032–40. doi: 10.3760/cma.j.cn112152-20230711-00289, PMID: 38110311

[23] Kang B Zhao ZQ Liu XY Cheng YX Tao W Wei ZQ. Effect of hypoalbuminemia on short-term outcomes after colorectal cancer surgery: A propensity score matching analysis. Front Nutr. (2022) 9:925086. doi: 10.3389/fnut.2022.925086, PMID: 36105581 PMC9464913

[24] Haskins IN Baginsky M Amdur RL Agarwal S. Preoperative hypoalbuminemia is associated with worse outcomes in colon cancer patients. Clin Nutr. (2017) 36:1333–8. doi: 10.1016/j.clnu.2016.08.023, PMID: 27612919

[25] Hu WH Eisenstein S Parry L Ramamoorthy S. Preoperative malnutrition with mild hypoalbuminemia associated with postoperative mortality and morbidity of colorectal cancer: a propensity score matching study. Nutr J. (2019) 18:33. doi: 10.1186/s12937-019-0458-y, PMID: 31253199 PMC6598281

[26] Zeng Q Li N Pan XF Chen L Pan A. Clinical management and treatment of obesity in China. Lancet Diabetes Endocrinol. (2021) 9:393–405. doi: 10.1016/S2213-8587(21)00047-4, PMID: 34022157

[27] Xue Z Yu J Higashikuchi T Compher C. Does low body mass index predict mortality in asian hospitalized patients. JPEN J Parenter Enteral Nutr. (2020) 44:722–8. doi: 10.1002/jpen.1708, PMID: 31556136

[28] Wang L Zhou B Zhao Z Yang L Zhang M Jiang Y. Body-mass index and obesity in urban and rural China: findings from consecutive nationally representative surveys during 2004-18. Lancet. (2021) 398:53–63. doi: 10.1016/S0140-6736(21)00798-4, PMID: 34217401 PMC7617101

[29] Japanese Gastric Cancer Association. Japanese gastric cancer treatment guidelines 2021 (6th edition). Gastric Cancer. (2023) 26:1–25. doi: 10.1007/s10120-022-01331-8, PMID: 36342574 PMC9813208

[30] Li J Zhang Y Hu DM Gong TP Xu R Gao J. Impact of postoperative complications on long-term outcomes of patients following surgery for gastric cancer: A systematic review and meta-analysis of 64 follow-up studies. Asian J Surg. (2020) 43:719–29. doi: 10.1016/j.asjsur.2019.10.007, PMID: 31703889

[31] Obana A Iwasaki K Suwa T. Impact of postoperative complications on gastric cancer survival. Surgery. (2025) 178:108873. doi: 10.1016/j.surg.2024.09.031, PMID: 39433448

[32] Kanda M Ito S Mochizuki Y Teramoto H Ishigure K Murai T. Multi-institutional analysis of the prognostic significance of postoperative complications after curative resection for gastric cancer. Cancer Med. (2019) 8:5194–201. doi: 10.1002/cam4.2439, PMID: 31353821 PMC6718595

[33] Kurita N Miyata H Gotoh M Shimada M Imura S Kimura W. Risk model for distal gastrectomy when treating gastric cancer on the basis of data from 33,917 Japanese patients collected using a nationwide web-based data entry system. Ann Surg. (2015) 262:295–303. doi: 10.1097/SLA.0000000000001127, PMID: 25719804

[34] Goglia M Pepe S Pace M Fattori L Minervini A Giulitti D. Complication of gastric cancer surgery: A single centre experience. In Vivo. (2023) 37:2166–72. doi: 10.21873/invivo.13315, PMID: 37652505 PMC10500523

[35] Lee CS Lee AY. Clinical applications of continual learning machine learning. Lancet Digit Health. (2020) 2:e279–279e281. doi: 10.1016/S2589-7500(20)30102-3, PMID: 33328120 PMC8259323

[36] De Bruyne S Speeckaert MM Van Biesen W Delanghe JR. Recent evolutions of machine learning applications in clinical laboratory medicine. Crit Rev Clin Lab Sci. (2021) 58:131–52. doi: 10.1080/10408363.2020.1828811, PMID: 33045173

[37] Zhang Y. Machine learning for health and clinical applications. Methods. (2022) 206:56–7. doi: 10.1016/j.ymeth.2022.08.004, PMID: 35964861 PMC9365712

[38] Li Z Bai B Zhao Y Yu D Lian B Liu Y. Severity of complications and long-term survival after laparoscopic total gastrectomy with D2 lymph node dissection for advanced gastric cancer: A propensity score-matched, case-control study. Int J Surg. (2018) 54:62–9. doi: 10.1016/j.ijsu.2018.04.034, PMID: 29698790

[39] Li P Huang CM Tu RH Lin JX Lu J Zheng CH. Risk factors affecting unplanned reoperation after laparoscopic gastrectomy for gastric cancer: experience from a high-volume center. Surg Endosc. (2017) 31:3922–31. doi: 10.1007/s00464-017-5423-2, PMID: 28205027

[40] Tang WZ Tan ZK Qiu LY Chen JQ Jia K. Prevalence and unfavorable outcome of frailty in older adults with gastric cancer: a systematic review and meta-analysis. Support Care Cancer. (2024) 32:115. doi: 10.1007/s00520-024-08306-8, PMID: 38240829

[41] Yang SJ Li HR Zhang WH Liu K Zhang DY Sun LF. Visceral fat area (VFA) superior to BMI for predicting postoperative complications after radical gastrectomy: a prospective cohort study. J Gastrointest Surg. (2020) 24:1298–306. doi: 10.1007/s11605-019-04259-0, PMID: 31161593

[42] Bacoeur-Ouzillou O Voron T Lambert C Fuks D Piessen G Manceau G. Impact of obesity on outcomes following surgery for gastric adenocarcinoma: A European multi-institutional study. Eur J Surg Oncol. (2024) 51:109518. doi: 10.1016/j.ejso.2024.109518, PMID: 39647445

[43] Chen HN Chen XZ Zhang WH Yang K Chen XL Zhang B. The impact of body mass index on the surgical outcomes of patients with gastric cancer: A 10-year, single-institution cohort study. Med (Baltimore). (2015) 94:e1769. doi: 10.1097/MD.0000000000001769, PMID: 26496304 PMC4620840

[44] Takeuchi M Ishii K Seki H Yasui N Sakata M Shimada A. Excessive visceral fat area as a risk factor for early postoperative complications of total gastrectomy for gastric cancer: a retrospective cohort study. BMC Surg. (2016) 16:54. doi: 10.1186/s12893-016-0168-8, PMID: 27494994 PMC4974690

[45] Matsui R Inaki N Tsuji T Kokura Y Momosaki R. Preoperative High Visceral Fat Increases Severe Complications but Improves Long-Term Prognosis after Gastrectomy for Patients with Advanced Gastric Cancer: A Propensity Score Matching Analysis. Nutrients. (2022) 14:4236. doi: 10.3390/nu14204236, PMID: 36296920 PMC9607456

[46] Shin HJ Son SY Cui LH Byun C Hur H Lee JH. Is there any role of visceral fat area for predicting difficulty of laparoscopic gastrectomy for gastric cancer. J Gastric Cancer. (2015) 15:151–8. doi: 10.5230/jgc.2015.15.3.151, PMID: 26468412 PMC4604329

[47] Miki Y Makuuchi R Tokunaga M Tanizawa Y Bando E Kawamura T. Risk factors for postoperative pneumonia after gastrectomy for gastric cancer. Surg Today. (2016) 46:552–6. doi: 10.1007/s00595-015-1201-8, PMID: 26077287

[48] Cilloniz C Torres A. Diabetes mellitus and pneumococcal pneumonia. Diagn (Basel). (2024) 14:859. doi: 10.3390/diagnostics14080859, PMID: 38667504 PMC11049506

[49] Jahan I Pandya J Munshi R Sen S. Glycocalyx disruption enhances motility, proliferation and collagen synthesis in diabetic fibroblasts. Biochim Biophys Acta Mol Cell Res. (2021) 1868:118955. doi: 10.1016/j.bbamcr.2021.118955, PMID: 33421533

[50] Horton WB Barrett EJ. Microvascular dysfunction in diabetes mellitus and cardiometabolic disease. Endocr Rev. (2021) 42:29–55. doi: 10.1210/endrev/bnaa025, PMID: 33125468 PMC7846151

[51] Mayhew D Mendonca V Murthy B. A review of ASA physical status - historical perspectives and modern developments. Anaesthesia. (2019) 74:373–9. doi: 10.1111/anae.14569, PMID: 30648259

[52] Nishibeppu K Sakuramoto S Matsui K Ebara G Fujita S Fujihata S. Dismal prognosis of elderly gastric cancer patients who underwent gastrectomy with American Society of Anesthesiologists (ASA) 3. Langenbecks Arch Surg. (2022) 407:3413–21. doi: 10.1007/s00423-022-02672-9, PMID: 36066671

[53] Park SH Eom SS Eom BW Yoon HM Kim YW Ryu KW. Postoperative complications and their risk factors of completion total gastrectomy for remnant gastric cancer following an initial gastrectomy for cancer. J Gastric Cancer. (2022) 22:210–9. doi: 10.5230/jgc.2022.22.e19, PMID: 35938367 PMC9359885

[54] Geroin C Weindelmayer J Camozzi S Leone B Turolo C Hetoja S. Clinical predictors of postoperative complications in the context of enhanced recovery (ERAS) in patients with esophageal and gastric cancer. Updates Surg. (2024) 76:1855–64. doi: 10.1007/s13304-023-01739-6, PMID: 38358642 PMC11455705

[55] Jiang Y Yang F Ma J Zhang N Zhang C Li G. Surgical and oncological outcomes of distal gastrectomy compared to total gastrectomy for middle-third gastric cancer: A systematic review and meta-analysis. Oncol Lett. (2022) 24:291. doi: 10.3892/ol.2022.13411, PMID: 35949603 PMC9353235

[56] Huang DD Wu GF Luo X Song HN Wang WB Liu NX. Value of muscle quality, strength and gait speed in supporting the predictive power of GLIM-defined malnutrition for postoperative outcomes in overweight patients with gastric cancer. Clin Nutr. (2021) 40:4201–8. doi: 10.1016/j.clnu.2021.01.038, PMID: 33583658

[57] Xu LB Shi MM Huang ZX Zhang WT Zhang HH Shen X. Impact of malnutrition diagnosed using Global Leadership Initiative on Malnutrition criteria on clinical outcomes of patients with gastric cancer. JPEN J Parenter Enteral Nutr. (2022) 46:385–94. doi: 10.1002/jpen.2127, PMID: 33908649

[58] Huang DD Yu DY Wang WB Song HN Luo X Wu GF. Global leadership initiative in malnutrition (GLIM) criteria using hand-grip strength adequately predicts postoperative complications and long-term survival in patients underwent radical gastrectomy for gastric cancer. Eur J Clin Nutr. (2022) 76:1323–31. doi: 10.1038/s41430-022-01109-2, PMID: 35314767

[59] Lin GT Huang JB Lin JL Lin JX Xie JW Wang JB. Body composition parameters for predicting the efficacy of neoadjuvant chemotherapy with immunotherapy for gastric cancer. Front Immunol. (2022) 13:1061044. doi: 10.3389/fimmu.2022.1061044, PMID: 36569876 PMC9772614

[60] Wang X Huang J Huang H Liu Y Ji C Liu J. Safety and efficacy of immunotherapy plus chemotherapy as neoadjuvant treatment for patients with locally advanced gastric cancer: a retrospective cohort study. Invest New Drugs. (2023) 41:579–86. doi: 10.1007/s10637-023-01379-y, PMID: 37368088

[61] Zhang X Zhang C Hou H Zhang Y Jiang P Zhou H. Neoadjuvant PD-1 blockade plus chemotherapy versus chemotherapy alone in locally advanced stage II-III gastric cancer: A single-centre retrospective study. Transl Oncol. (2023) 31:101657. doi: 10.1016/j.tranon.2023.101657, PMID: 36934638 PMC10034143

