# Supplementary material for: Development and validation of a machine learning model to predict postoperative complications following radical gastrectomy for gastric cancer
Source: Front Oncol. 2025 Sep 8;15:1606938. doi: 10.3389/fonc.2025.1606938 (PMC12450669; doi:10.3389/fonc.2025.1606938)
Supplement: Supplementary file 1 [file DataSheet1.doc]

**SUPPLEMENTARY TABLE 1 Univariate analysis of variables associated with postoperative complications in the t**raining cohort

| Variable | No complication (n=1182) | Complication (n=304) | p |
| --- | --- | --- | --- |
| Age (years) |  |  | 0.002 |
| <65 | 703 (59.5) | 151 (49.7) |  |
| ≥65 | 479 (40.5) | 153 (50.3) |  |
| Sex |  |  | 0.613 |
| Male | 788 (66.7) | 198 (65.1) |  |
| Female | 394 (33.3) | 106 (34.9) |  |
| BMI (kg/m^2^) |  |  | <0.001 |
| underweight | 78 ( 6.6) | 14 ( 4.6) |  |
| normal | 836 (70.7) | 181 (59.5) |  |
| overweight | 268 (22.7) | 109 (35.9) |  |
| Previous abdominal surgery |  |  | 0.105 |
| Yes | 198 (16.8) | 63 (20.7) |  |
| No | 984 (83.2) | 241 (79.3) |  |
| Drinking history |  |  | 0.268 |
| Yes | 320 (27.1) | 92 (30.3) |  |
| No | 862 (72.9) | 212 (69.7) |  |
| Smoking history |  |  | 0.091 |
| Yes | 279 (23.6) | 86 (28.3) |  |
| No | 903 (76.4) | 218 (71.7) |  |
| Diabetes mellitus |  |  | 0.007 |
| Yes | 218 (18.4) | 77 (25.3) |  |
| No | 964 (81.6) | 227 (74.7) |  |
| Hypertension |  |  | 0.083 |
| Yes | 296 (25.0) | 91 (29.9) |  |
| No | 886 (75.0) | 213 (70.1) |  |
| Dyslipidemia |  |  | 0.489 |
| Yes | 277 (23.4) | 77 (25.3) |  |
| No | 905 (76.6) | 227 (74.7) |  |
| Tumor location |  |  | 0.056 |
| Upper-third | 314 (26.6) | 80 (26.3) |  |
| Middle-third | 353 (29.9) | 111 (36.5) |  |
| Lower-third | 515 (43.6) | 113 (37.2) |  |
| FEV1/FVC, %, median (IQR) | 72.00 [69.00, 73.00] | 72.00 [68.00, 73.00] | 0.086 |
| Tumor size (cm) |  |  | 0.145 |
| <5 | 654 (55.3) | 154 (50.7) |  |
| ≥5 | 528 (44.7) | 150 (49.3) |  |
| ASA grade |  |  | 0.001 |
| I | 859 (72.7) | 191 (62.8) |  |
| II | 223 (18.9) | 68 (22.4) |  |
| III | 100 ( 8.5) | 45 (14.8) |  |
| Neoadjuvant chemotherapy |  |  | 0.039 |
| Yes | 300 (25.4) | 95 (31.2) |  |
| No | 882 (74.6) | 209 (68.8) |  |
| Multivisceral resection |  |  | 0.041 |
| Yes | 61 (5.2) | 25 (8.2) |  |
| No | 1121 (94.8) | 279 (91.8) |  |
| Preoperative anemia |  |  | 0.078 |
| Yes | 354 (29.9) | 107 (35.2) |  |
| No | 828 (70.1) | 197 (64.8) |  |
| Preoperative hypoalbuminemia |  |  | 0.088 |
| Yes | 264 (22.3) | 82 (27.0) |  |
| No | 918 (77.7) | 222 (73.0) |  |
| Preoperative WBC,×10^9^/L, median (IQR) | 6.50 [5.40, 7.60] | 6.40 [5.30, 7.23] | 0.169 |
| Preoperative BUN, mg/dl, median (IQR) | 6.45 [5.67, 7.24] | 6.67 [5.67, 7.45] | 0.246 |
| Preoperative total bilirubin, mg/dl, median (IQR) | 12.85 [9.70, 16.90] | 13.80 [10.55, 17.00] | 0.300 |
| CEA, ng/ml |  |  | 0.483 |
| <5 | 943 (79.8) | 248 (81.6) |  |
| ≥5 | 239 (20.2) | 56 (18.4) |  |
| CA19-9, U/ml |  |  | 0.214 |
| <30 | 1017 (86.0) | 253 (83.2) |  |
| ≥30 | 165 (14.0) | 51 (16.8) |  |
| Surgical approach |  |  | 0.130 |
| Open | 359 (30.4) | 78 (25.7) |  |
| Laparoscopic | 823 (69.6) | 226 (74.3) |  |
| Operation time (h) |  |  | 0.001 |
| <3 | 626 (53.0) | 128 (42.1) |  |
| ≥3 | 556 (47.0) | 176 (57.9) |  |
| Estimated blood loss (ml) |  |  | 0.159 |
| <200 | 551 (46.6) | 128 (42.1) |  |
| ≥200 | 631 (53.4) | 176 (57.9) |  |
| Histological type |  |  | 0.735 |
| Well/ Moderately | 291 (24.6) | 72 (23.7) |  |
| Poorly/ Undifferentiated | 891 (75.4) | 232 (76.3) |  |
| Type of operation |  |  | 0.001 |
| Distal gastrectomy | 704 (59.6) | 145 (47.7) |  |
| Proximal gastrectomy | 59 (5.0) | 17 (5.6) |  |
| Total gastrectomy | 419 (35.4) | 142 (46.7) |  |
| Extent of lymph node dissection |  |  | 0.719 |
| < D2 | 141 (11.9) | 34 (11.2) |  |
| ≥ D2 | 1041 (88.1) | 270 (88.8) |  |
| Intraoperative blood transfusion |  |  | 0.136 |
| Yes | 198 (16.8) | 62 (20.4) |  |
| No | 984 (83.2) | 242 (79.6) |  |
| Reconstruction method |  |  | 0.367 |
| Intracorporeal | 119 (10.1) | 36 (11.8) |  |
| Extracorporeal | 1063 (89.9) | 268 (88.2) |  |
| Number of removed lymph nodes, mean (SD) | 34.3±13.0 | 32.8±13.0 | 0.065 |
| Pathological stage |  |  | 0.131 |
| I | 270 (22.8) | 79 (26.0) |  |
| II | 192 (16.2) | 59 (19.4) |  |
| III | 720 (60.9) | 166 (54.6) |  |

BMI, body mass index; ASA, American society of anesthesiologists classification; WBC, white blood cell; BUN, blood urea nitrogen; CEA, carcinoembryonic antigen; CA19-9, cancer antigen 19-9; IQR, interquartile range; SD, standard deviation.

**SUPPLEMENTARY TABLE 2 Univariate analysis of variables associated with postoperative complications in the v**alidation cohort

| Variable | No complication (n= 379) | Complication  (n= 119) | p |
| --- | --- | --- | --- |
| Age (years) |  |  | 0.001 |
| <65 | 226 (59.6) | 51 (42.9) |  |
| ≥65 | 153 (40.4) | 68 (57.1) |  |
| Sex |  |  | 0.262 |
| Male | 278 (73.4) | 81 (68.1) |  |
| Female | 101 (26.6) | 38 (31.9) |  |
| BMI (kg/m^2^) |  |  | 0.015 |
| underweight | 36 ( 9.5) | 2 ( 1.7) |  |
| normal | 255 (67.3) | 83 (69.7) |  |
| overweight | 88 (23.2) | 34 (28.6) |  |
| Previous abdominal surgery |  |  | 0.767 |
| Yes | 78 (20.6) | 23 (19.3) |  |
| No | 301 (79.4) | 96 (80.7) |  |
| Drinking history |  |  | 0.116 |
| Yes | 79 (20.8) | 33 (27.7) |  |
| No | 300 (79.2) | 86 (72.3) |  |
| Smoking history |  |  | 0.289 |
| Yes | 93 (24.5) | 35 (29.4) |  |
| No | 286 (75.5) | 84 (70.6) |  |
| Diabetes mellitus |  |  | 0.007 |
| Yes | 306 (80.7) | 82 (68.9) |  |
| No | 73 (19.3) | 37 (31.1) |  |
| Hypertension |  |  | 0.407 |
| Yes | 103 (27.2) | 37 (31.1) |  |
| No | 276 (72.8) | 82 (68.9) |  |
| Dyslipidemia |  |  | 0.152 |
| Yes | 115 (30.3) | 28 (23.5) |  |
| No | 264 (69.7) | 91 (76.5) |  |
| Tumor location |  |  | 0.290 |
| Upper-third | 105 (27.7) | 41 (34.5) |  |
| Middle-third | 121 (31.9) | 38 (31.9) |  |
| Lower-third | 153 (40.4) | 40 (33.6) |  |
| FEV1/FVC, %, median (IQR) | 72.00 [68.00, 73.00] | 72.00 [69.00, 75.00] | 0.131 |
| Tumor size (cm) |  |  | 0.219 |
| <5 | 206 (54.4) | 57 (47.9) |  |
| ≥5 | 173 (45.6) | 62 (52.1) |  |
| ASA grade |  |  |  |
| I | 275 (72.6) | 59 (49.6) | <0.001 |
| II | 71 (18.7) | 33 (27.7) |  |
| III | 33 ( 8.7) | 27 (22.7) |  |
| Neoadjuvant chemotherapy |  |  | <0.001 |
| Yes | 98 (25.9) | 52 (43.7) |  |
| No | 281 (74.1) | 67 (56.3) |  |
| Multivisceral resection |  |  | 0.014 |
| Yes | 20 ( 5.3) | 14 (11.8) |  |
| No | 359 (94.7) | 105 (88.2) |  |
| Preoperative anemia |  |  | 0.119 |
| Yes | 114 (30.1) | 27 (22.7) |  |
| No | 265 (69.9) | 92 (77.3) |  |
| Preoperative hypoalbuminemia |  |  | 0.051 |
| Yes | 90 (23.7) | 39 (32.8) |  |
| No | 289 (76.3) | 80 (67.2) |  |
| Preoperative WBC,×10^9^/L, median (IQR) | 6.30 [5.30, 7.20] | 6.50 [5.40, 7.80] | 0.101 |
| Preoperative BUN, mg/dl, median (IQR) | 6.45 [5.75, 7.24] | 6.76 [5.71, 7.56] | 0.126 |
| Preoperative total bilirubin, mg/dl, median (IQR) | 13.40 [10.80, 17.30] | 13.90 [10.90, 17.50] | 0.618 |
| CEA, ng/ml |  |  | 0.895 |
| <5 | 294 (77.6) | 93 (78.2) |  |
| ≥5 | 85 (22.4) | 26 (21.8) |  |
| CA19-9, U/ml |  |  | 0.177 |
| <30 | 304 (80.2) | 102 (85.7) |  |
| ≥30 | 75 (19.8) | 17 (14.3) |  |
| Surgical approach |  |  | 0.090 |
| Open | 106 (28.0) | 43 (36.1) |  |
| Laparoscopic | 273 (72.0) | 76 (63.9) |  |
| Operation time (h) |  |  | 0.040 |
| <3 | 197 (52.0) | 49 (41.2) |  |
| ≥3 | 182 (48.0) | 70 (58.8) |  |
| Estimated blood loss (ml) |  |  | 0.180 |
| <200 | 173 (45.6) | 46 (38.7) |  |
| ≥200 | 206 (54.4) | 73 (61.3) |  |
| Histological type |  |  | 0.524 |
| Well/ Moderately | 87 (23.0) | 24 (20.2) |  |
| Poorly/ Undifferentiated | 292 (77.0) | 95 (79.8) |  |
| Type of operation |  |  | <0.001 |
| Distal gastrectomy | 254 (67.0) | 53 (44.5) |  |
| Proximal gastrectomy | 22 ( 5.8) | 4 ( 3.4) |  |
| Total gastrectomy | 103 (27.2) | 62 (52.1) |  |
| Extent of lymph node dissection |  |  | 0.234 |
| < D2 | 31 ( 8.2) | 14 (11.8) |  |
| ≥ D2 | 348 (91.8) | 105 (88.2) |  |
| Intraoperative blood transfusion |  |  | 0.262 |
| Yes | 54 (14.2) | 22 (18.5) |  |
| No | 325 (85.8) | 97 (81.5) |  |
| Reconstruction method |  |  | 0.384 |
| Intracorporeal | 40 (10.6) | 16 (13.4) |  |
| Extracorporeal | 339 (89.4) | 103 (86.6) |  |
| Number of removed lymph nodes, mean (SD) | 31.96±12.40 | 33.46±13.24 | 0.257 |
| Pathological stage |  |  | 0.236 |
| I | 81 (21.4) | 32 (26.9) |  |
| II | 55 (14.5) | 21 (17.6) |  |
| III | 243 (64.1) | 66 (55.5) |  |

BMI, body mass index; ASA, American society of anesthesiologists classification; WBC, white blood cell; BUN, blood urea nitrogen; CEA, carcinoembryonic antigen; CA19-9, cancer antigen 19-9; IQR, interquartile range; SD, standard deviation.


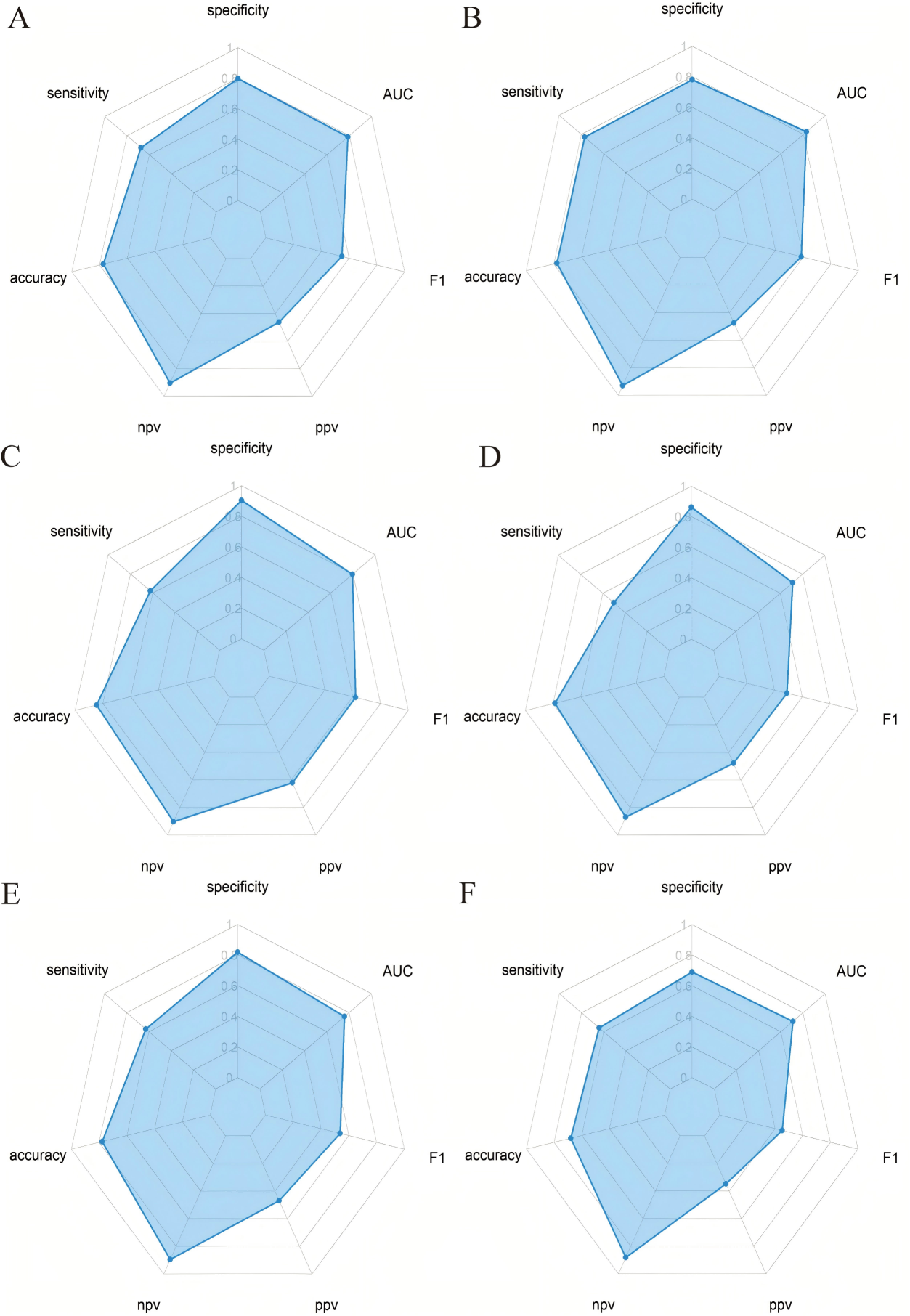


**SUPPLEMENTARY FIGURE 1**

Radar plots of machine learning models in training cohort: (A)TB, (B) RF, (C) SVM, (D) XGBoost, (E) GNB, (F) ANN.

**SUPPLEMENTARY TABLE 3 Performance of ML Models in Validation Cohort.**

|  | AUC  (95%CI) | Accuracy  (95%CI) | Sensitivity (95%CI) | Specificity (95%CI) | PPV  (95%CI) | NPV  (95%CI) | F1 score (95%CI) |
| --- | --- | --- | --- | --- | --- | --- | --- |
| TB | 0.859  (0.821-0.896) | 0.797  (0.763-0.828) | 0.856  (0.811-0.892) | 0.778  (0.736-0.806) | 0.546  (0.508-0.584) | 0.946  (0.908-0.989) | 0.667  (0.627-0.704) |
| RF | 0.871  (0.833-0.908) | 0.833  (0.801-0.867) | 0.729  (0.691-0.761) | 0.865  (0.827-0.905) | 0.628  (0.589-0.661) | 0.911  (0.879-0.942) | 0.675  (0.625-0.721) |
| SVM | 0.806  (0.760-0.852) | 0.795  (0.761-0.829) | 0.686  (0.642-0.721) | 0.828  (0.795-0.867) | 0.555  (0.508-0.602) | 0.895  (0.858-0.931) | 0.614  (0.584-0.658) |
| XGBoost | 0.724  (0.672-0.777) | 0.676  (0.621-0.728) | 0.729  (0.684-0.763) | 0.660  (0.639-0.693) | 0.400  (0.365-0.440) | 0.887  (0.853-0.917) | 0.517  (0.487-0.547) |
| GNB | 0.756  (0.700-0.811) | 0.761  (0.721-0.803) | 0.653  (0.621-0.693) | 0.794  (0.758-0.832) | 0.497  (0.462-0.532) | 0.880  (0.848-0.923) | 0.564  (0.517-0.607) |
| ANN | 0.777  (0.733-0.820) | 0.616  (0.582-0.648) | 0.941  (0.902-0.978) | 0.515  (0.487-0.574) | 0.376  (0.342-0.405) | 0.965  (0.932-0.995) | 0.537  (0.489-0.563) |


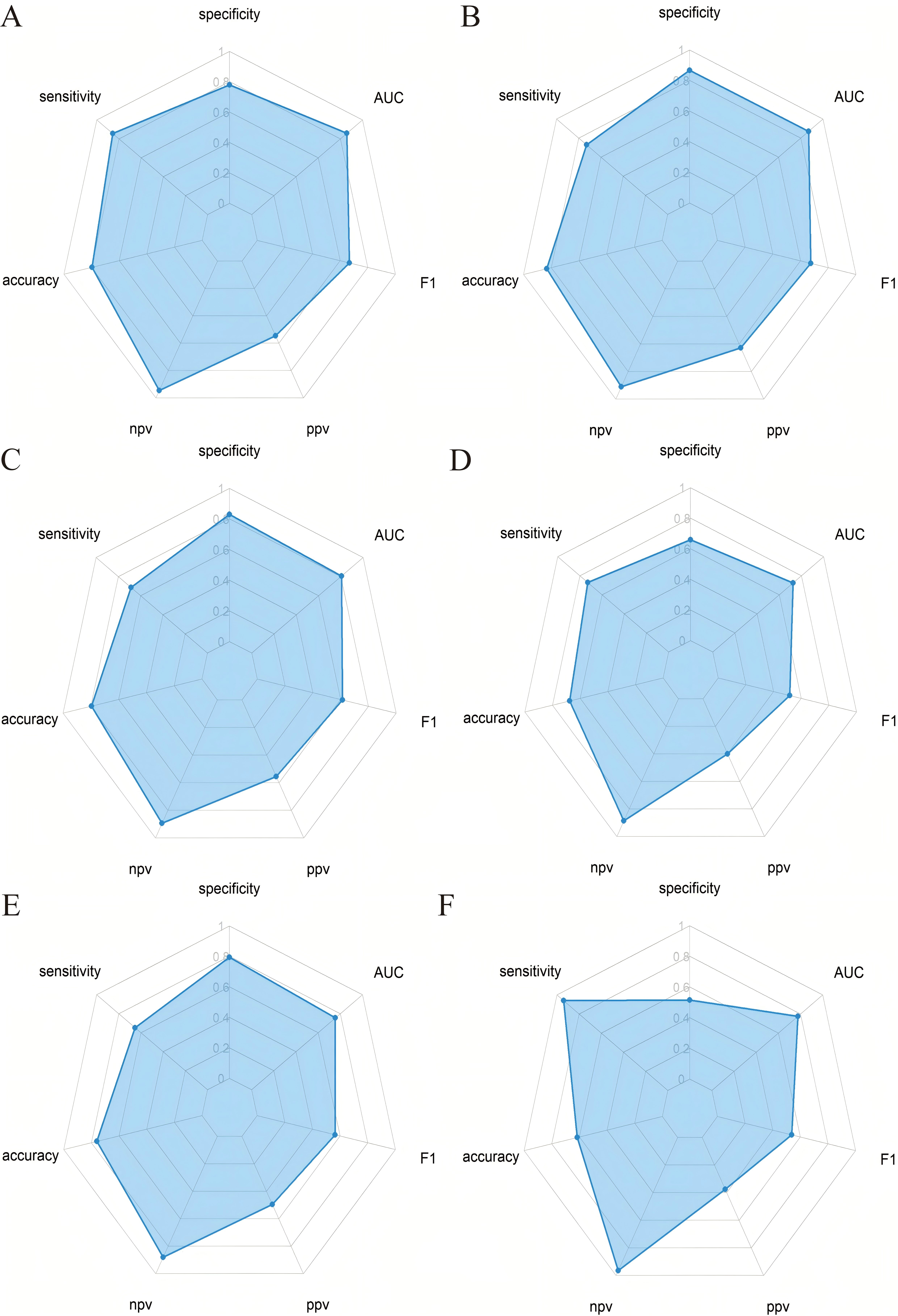


**SUPPLEMENTARY FIGURE 2**

Radar plots of machine learning models in validation cohort: (A) TB, (B) RF, (C) SVM, (D) XGBoost, (E) GNB, (F) ANN.
